# Re-surgery and chest wall re-irradiation for recurrent breast cancer - a second curative approach

**DOI:** 10.1186/1471-2407-11-197

**Published:** 2011-05-25

**Authors:** Arndt-Christian Müller, Franziska Eckert, Vanessa Heinrich, Michael Bamberg, Sara Brucker, Thomas Hehr

**Affiliations:** 1 Department of Radiooncology, Eberhard-Karls-University, Hoppe-Seyler-Str. 3, 72076 Tübingen, Germany; 2 Department of Gynecology, Eberhard-Karls-University, Calwerstr. 7, 72076 Tübingen, Germany; 3 Department of Radiooncology, Marienhospital Stuttgart, Böheimstr. 37, 70199 Stuttgart, Germany

## Abstract

**Background:**

Repeat radiation is a rarely used treatment strategy that must be performed with caution. The efficacy and toxicity of a second curative radiotherapy series was investigated in cases of recurrent breast cancer.

**Methods:**

Forty-two patients treated from 1993 to 2003 with resection (n = 30) and postoperative re-irradiation or definitive re-irradiation (n = 12) for recurrent breast cancer were enrolled in the study. Concurrent hyperthermia was performed in 29 patients. The median age was 57 years. The median pre-radiation exposure was 54Gy. Re-irradiation was conventionally fractionated to a median total dose of 60Gy.

**Results:**

After a median follow-up of 41 months (range 3-92 months) higher graded late toxicity > G3 according to CTC 3.0 and LENT-SOMA was not observed. The estimated 5-year local control rate reached 62%. The estimated 5-year overall survival rate was 59%. Significantly inferior survival was associated with recurrence within two years (40 vs. 71%, p < ([0-9]).01) and presence of macroscopic tumour load (24 vs. 75%, p = 0.03).

**Conclusions:**

Repeat radiotherapy for recurrent breast cancer with total radiation doses of 60 Gy and the addition of hyperthermia in the majority of patients was feasible, with acceptable late morbidity and improved prognosis, particularly in patients with previous resection of recurrent tumours.

## Background

Treatment of pre-radiated regions remains a therapeutic challenge for breast cancer, particularly as the curative potential of local (surgery, radiotherapy) and systemic (anti-hormonal therapy, chemotherapy) treatment of loco-regional recurrences has yet to be elucidated [1,2]. After treatment with breast-conserving surgery and radiotherapy, the standard procedure is to carry out salvage mastectomy for in-breast recurrences [3]. In highly selected patients with in-breast tumours (< 3 cm, no skin or chest wall infiltration, cN0, cM0) a skin sparing mastectomy with immediate breast reconstruction is a possible treatment option with a local control rate of 90% [4]. In contrast, in lesser selected series, mastectomy for invasive recurrences causes local control to drop to 68-87% [5].

Treating chest wall recurrences after mastectomy with re-surgery alone provides limited local control for one-third of patients [6]. The routine addition of a second radiotherapy series is accompanied by major concerns regarding side effects, as cumulative radiation doses reach or exceed 100 Gy, which may lead to an increased rate of late complications. However, combined treatment of chest wall recurrences i.e. excision plus irradiation revealed superior local control of 48 vs. 34% after five years in single institution series [7]. For irresectable recurrences, re-irradiation combined with additional hyperthermia enhanced efficacy i.e. local control compared to radiotherapy alone by approximately 20% [8,9]. On the basis of the results from these prospective trials, thermoradiotherapy has become a standard treatment for patients with irresectable recurrent lesions particularly those in previously irradiated areas.

No definitive trial investigating the options of re-treatment i.e. re-irradiation +/- re-surgery has been carried out, and is never likely to be done in view of the aforementioned concerns. Unequal pre-radiation exposure (40-70 Gy) would lead to considerable different risks of late effects within the study population and distort results. Therefore, this retrospective study was performed to evaluate the long-term results and toxicity of local treatment by re-irradiation +/- preceding macroscopically complete resection with curative intent. Furthermore, this analysis focused on tumour- and treatment-related parameters to optimize management of breast cancer recurrences in the future.

## Methods

Between 1993 and 2003, 42 women with locoregional recurrent breast cancer were treated with a macroscopically complete resection plus subsequent re-irradiation of the chest wall (n = 30) or definitive radiotherapy (n = 12) at the University of Tübingen.

For inclusion in this retrospective analysis, patients had to fulfill the following criteria: All patients had been initially treated for invasive breast cancer using mastectomy or breast conserving surgery, both combined with adjuvant radiotherapy of the whole breast or the chest wall including regional lymphatics, or a radiation boost if indicated. Patients with preceding or simultaneous metastases, new primary tumours unrelated to their breast cancer or receiving palliative treatment were excluded. The patients fulfilling these criteria were identified by the departmental database. After discussing the intended analysis, the institutional review board (Ethikkommision der Medizinischen Fakultät, Gartenstrasse 47, 72074 Tübingen) had no objections (Nr. 116/2011A).

A brief summary of the clinical characteristics at first diagnosis of breast cancer is provided in Table 1. The first session of radiotherapy was carried out using 6 MV photons with tangential fields or 4-12 MeV electrons with one/multiple fields or an electron rotation. Gamma irradiation with cobalt 60 was rarely administered. Field definitions were standard definitions of the treatment time as published by Sack and Thesen [10]. The median pre-radiation exposure was 54 Gy.

**Table 1:** Tumour- and treatment characteristics at initial diagnosis.

Characteristic at initial diagnosis	Value
**Patients (n)**	42

**Year of first treatment**	
Median	1992
Range	1968-2002

**Age (y)**	
Range	30-66
Median	49

**pT-stage (n)**	
x	1
1	15
2	17
3	7
4	2

**pN-status (n)**	
axillary lymph node dissection	41
node-negative	22
node-positive	19
unknown	1

**pR-status (n)**	
R0	31
R1	7
R2	1
Rx	3

**Grading (n)**	
I	5
II	23
III	9
unknown	5

**Hormone receptor expression (n)**	
ER-positive	21
PR-positive	19
unknown	3

**Surgery of primary breast cancer (n)**	
Mastectomy	18
Breast conserving surgery	24

**Radiation treatment of primary breast cancer(n)**	
Adjuvant total radiation breast/chest wall dose (Gy)	
Median	54
Range	40-65.4
Lymph node irradiation	25

**Systemic treatment of primary breast cancer (n)**	
Chemotherapy	24
Hormonal treatment	10

In-breast recurrences were treated by mastectomy. Chest wall recurrences were removed by local excision- if possible. Re-irradiation was not routinely performed in cases of resected recurrence as an "adjuvant" procedure. Preconditions of individual re-irradiation were close (≤0.5 cm) or positive margins, perinodal involvement, multiple recurrences or other high-risk features explaining a number of recurrences totaling five until re-irradiation was administered.

Details regarding second treatments and the course of disease at the time of recurrence are displayed in Table 2. The median time to local recurrence calculated from the first radiotherapy session was 33 months and ranged between 9 and 400 months. The median time between the two radiation courses was 53 months. Re-irradiation was conventionally fractionated with single doses of 1.8-2.0 Gy five times/week (in one individual case 4 × 2.5 Gy/week), resulting in a median total dose of 60 Gy, including a fractionated boost of 4-16 Gy in 74% of women. Regional lymphatics (supra-/infraclavicular fossa, and if indicated parasternal or axillary nodes) were covered by radiation portals in 15 patients (36%) to a median total dose of 50 Gy. Parasternal nodes were irradiated in seven patients (17%) using the mixed-beam-technique. Re-irradiation was performed using 6 MV photons (n = 24) with tangential fields or 4-12 MeV electrons with an electron beam rotation technique (n = 18) as described elsewhere [11]. Chestwall was defined as thoracic wall extending from the second to the sixth/seventh intercostal space including a small portion of the underlying lung. Longitudinal field borders were orientated to the position of the contralateral breast (inferior/superior margin 2 cm below palpable contralateral breast; the contralateral breast was not irradiated.). Medial margin was 1 cm over midline. Lateral margin was usually near midaxillary line. Resulting mean re-irradiation field size was 17 × 17 cm (ranging from 8 × 8 cm to 35 × 20 cm). In most cases, the whole chestwall was covered (n = 34) according to the above mentioned target volume definition. Eight patients were treated with involved field techniques with a lateral margin of ≥3 cm. The brachial plexus and regional lymphatics were not re-irradiated.

**Table 2:** Patient characteristics at second radiation course.

Characteristic at diagnosis of recurrence	Value
**Patients (n)**	42

**Age (y)**	

Median	57
Range	33-75

**Time to first recurrence (months)**	
Median	33
Range	9-400

**Number of recurrences until re-irradiation**	
1	23
2	13
more than 2 (range)	6 (3-5)

**Site of recurrence (n)**	
Chest wall	31
In-breast recurrence	8
Regional nodal	3

**Surgery of recurrence preceeding re-irradiation (n)**	
Mastectomy	9*
Local excision	22
Inoperable	11

**pR-status for operable patients (n)**	
R0	14
R1	16
R2	1

**Re-Radiation treatment of recurrence**	
Total radiation chest wall dose (Gy)	
Median dose	60
Range	45-66
Lymph node irradiation (n)	15^#^
Cumulative radiation dose (Gy)	
Median dose	110
Range	85-126
Concurrent hyperthermia (n)	29
Photons (n)	24
Electrons (n)	18

**Time between both radiation courses (months)**	
Median	53
Range	12-401

**Systemic treatment of recurrent breast cancer (n)**	
Sequential chemotherapy	17
Hormonal treatment	19

*15 Salvage mastectomies were performed at first recurrence explaining the lower number of mastectomies at time of re-irradiation.

^#^Lymph node irradiation was normally restricted to sites initially not irradiated, i.e. axillary nodes.

In cases of close or positive margins and definitive treatment, superficial radiofrequency hyperthermia was offered as an additional modality, leading to concurrent application in 29 patients (69%). Almost all patients with microscopically positive margins received hyperthermia (n = 14; 88%), while after R0-resection (n = 8; 57%) or in cases of inoperable recurrences (n = 7; 58%), approximately three-fifths of patients received hyperthermia. Superficial hyperthermia was performed with the SA-115 applicator twice a week using a BSD2000 hyperthermia system operating at a frequency between 210-219 MHz. Median hyperthermia time was 75 min, i.e. 5-15 min heating period and at least 60 min treatment time (steady state of temperature). The target temperature was a minimum of 40°C and measured at the skin.

Hyperthermia was performed in accordance with the ESHO-Guidelines. A detailed description of hyperthermia with SA-115 has been published [12]. Further treatment before or after repeat radiotherapy consisted of sequential chemotherapy or hormonal therapy (Table 2).

After re-treatment, senological examinations were performed every three to six months during the first two years and every six to twelve months thereafter. These investigations included clinical examination and imaging if required. Patients were monitored at least annually in terms of toxicity by a radiation oncologist. The Patient's records and questionnaires were reviewed with respect to radiation-induced side effects. Acute radiation dermatitis was investigated during and after re-irradiation and documented according to CTC 3.0. Late toxicity was defined as side effects occurring three months after treatment. Radiation dermatitis, induration/fibrosis, teleangiectasia, pericarditis, pericardial effusion and pneumonitis were investigated according to CTC 3.0. In addition, side effects related to skin (fibrosis/teleangiectasia) and lung (fibrosis) were documented according to LENT-SOMA[13].

Local failure was defined as any recurrence of tumour in the ipsilateral chest wall or in mastectomy scars. Regional failure was defined as any recurrence of tumour in the ipsilateral regional nodes. Recurrence at any other site was considered as distant failure. Time to any failure until re-irradiation was defined as the time from definitive surgery to the time of diagnosis of the first failure. Time to any failure after the second radiation course was defined as the time from start of re-irradiation, in order to compare patients with and without excision.

Local control, distant-disease-free survival, disease-free survival and overall survival were the main endpoints, and these were calculated from time of re-irradiation using the Kaplan-Meier method [14]. Further subgroup analyses were performed for local control and overall survival. Initial tumour parameters (nodal stage, estrogen receptor status, time to first recurrence (≤2 years vs. > 2years), number of recurrences until re-irradiation (one vs. more than one)), surgery of recurrence, margin status, concurrent hyperthermia, lymph node irradiation, sequential chemotherapy and anti-hormonal therapy were investigated as factors. Actuarial curves were compared using the two-tailed log-rank test. A p-value of ≤0.05 was considered significant. Statistical analyses were performed using the software package SPSS 15.0 (SPSS Inc., Chicago, Illinois, USA).

## Results

The median follow-up from second radiation treatment was 41 months (range 3-92 months). One patient was lost to follow-up. For the 28 surviving women, the median follow-up reached 48 months. With respect to total dose, patients completed radiation treatment as planned. For one patient, a treatment break of two weeks was required owing to acute radiation dermatitis. Concurrent hyperthermia was offered to all patients but some had contraindications (thrombosis, cardiac insufficiency and hypertension) and others refused hyperthermia, leading to an omission rate of 31%. Hyperthermia was completed as planned in 72% of patients (n = 21/29). In two patients, the number of planned hyperthermia treatments was not given. In six patients, the duration of one hyperthermia treatment in each case was shorter than 60 min. Repeat radiotherapy was performed at first recurrence in median but 19 patients experienced at least two recurrences until re-irradiation was administered. Therefore, the median time to second radiotherapy (53 months) was 20 months longer than time to first recurrence (Table 2).

**Figure 1:**
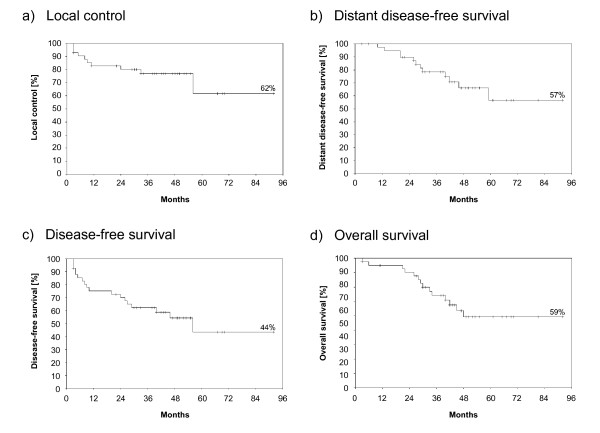
**Local control and survival parameters**. Local control (a), distant disease-free survival (b), disease-free survival (c) and overall survival were estimated using the Kaplan-Meier-method after a median follow-up of 41 months. The parameters were calculated from time of re-irradiation for recurrence.

Local and distant control and disease-free and overall-survival are presented in Figure 1a-d. The estimated five-year local control reached 62% (n = 32/42). Local failures occurred in 10 patients (R0: 3/14, R1: 3/16; R2/irresectable: 4/12). All patients with irresectable disease achieved a clinical complete remission for at least three months in the re-treated area.

Endocrine therapy increased local control (93 vs. 31%, p = 0.01). However, local control decreased significantly from 91% to 31% (p = 0.02) if at least two recurrences were experienced before re-irradiation was administered. A time period of less than two years to the first recurrence (< 2 years vs. ≥2years: 16 vs. 68%, p = 0.14) did not significantly lower local control. However, long-term local control at last follow-up improved to 70% (n = 35/42) owing to curative resections of recurrences after re-irradiation in three patients. In one of the three patients additional brachytherapy was performed to a total dose of 30 Gy. A sub-analysis of concurrent hyperthermia for R1-resected patients revealed a prolonged local control (86 vs. 50%, p = 0.19; with one salvage treatment after re-irradiation 93 vs. 50%, p = 0.05) but this was not significant. No other investigated factors significantly affected local control (data not shown).

**Figure 2:**
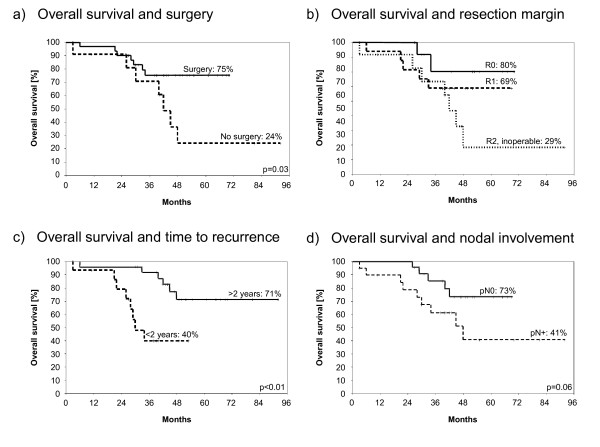
**Impact of treatment- and tumour-related parameters on overall survival**. Overall survival was significantly influenced by surgery of recurrence (a) and resection margin (b). R0-resection led to prolonged overall survival in contrast to patients with gross residual disease (80% vs. 29%, p = 0.04). Furthermore, time to first recurrence less than two years was associated with a significant detrimental effect on overall survival (c). A trend to prolonged overall survival was detected in patients without nodal involvement at initial diagnosis of breast cancer (d). All parameters were calculated from time of re-irradiation for recurrence.

The estimated five year overall survival of patients was 59%. Estimated overall survival was significantly shortened in patients who had not undergone surgery of recurrence (75 vs. 24%, p = 0.03), Figure 2a. Clear resection margins (R0) led to significantly prolonged overall survival in contrast to gross residual disease (80% vs. 29%, p = 0.0448). A trend to improved overall survival was observed for patients with R1-resection compared with patients with macroscopical residual disease, Figure 2b (69 vs. 29%, p = 0.06). Concurrent hyperthermia was associated by an increase of overall survival (67 vs. 37%, p = 0.27) but this was not significant.

Furthermore, the relevance of tumour-related parameters was investigated. Significantly poorer overall survival was detected in women (n = 15) where a time period of less than two years had passed before the first recurrence (71 vs. 40%, p < 0.01), Figure 2c. A trend to reduced overall survival was evident in women who had not been re-irradiated at first recurrence (75 vs. 43%; p = 0.11). Re-irradiation at second recurrence (n = 13) resulted in the lowest estimated survival curve with 34% overall survival compared to 75% overall survival at first recurrence (n = 23) (p = 0.08, data not shown). A trend to inferior survival was observed for initially node-positive patients (73 vs. 41%, p = 0.06), Figure 2d. Grading did not significantly lower outcome (G1 vs. G3: 80% to 37%, p = 0.37).

Regional (n = 3) and distant (n = 12) metastases manifested in 15 patients.. The mean distant disease-free survival time exceeded more than five years (68 months, median not reached, confidence interval (CI): 46-71 months). The mean disease-free survival was reached before 5 years (54 months, CI: 41-67 months).

No treatment related deaths occurred. Acute and late skin toxicity of radiation schedules are documented in Table 3. Grade 2 acute skin reactions of were doubled (12 vs. 25 patients) at the second radiotherapy session while acute grade 3 toxicity was inversely distributed (five vs. two patients). Grade 2 and 3 late skin toxicity of was elevated by approximately 12% after repeat irradiation (G2: 50 vs. 62%; G3:7 vs. 19%). Radiation-induced pneumonitis was observed after re-irradiation in four patients (twice grade 1 and 2) in contrast to one case of pneumonitis (grade 1) during the first radiation course. One patient developed rib-fractures. Brachial plexopathy, or pericarditis related to treatment were not observed. Therefore, no patient experienced toxicity higher than grade 3.

**Table 3:** Acute and late skin toxicity.

CTC-Grade	Acute skin toxicity		Late skin toxicity	
	1st radiotherapy	2nd radiotherapy	1st radiotherapy	2nd radiotherapy
0	2	0	4	1
1	21	15	14	7
2	12	25	21	26
3	5	2	3	8
4	0	0	0	0
unknown	2	0	0	0

The treatment-related cumulative cutaneous side effects were graded according to CTC 3.0 and documented for both radiation courses. Since the first radiation treatment was performed before the definition of CTC criteria, all data from medical charts were reviewed and converted into CTC 3.0 criteria to compare maximal occurred cumulative toxicity at first and second radiation courses. Maximal cumulated late toxicity of the first radiation course was assessed at last follow-up before treatment or at baseline examination before re-treatment.

## Discussion

Local breast cancer recurrences are a therapeutic challenge with respect to available treatment options, morbidity and toxicity. Depending on the risk features, local recurrence might further herald distant metastasis [15]. The optimal treatment procedures have been debated for at least two decades [2,16,17]. The complexity of treatment increases, particularly in patients who have previously undergone radiotherapy, as total re-treatment doses below 55 Gy produce poor local control rates without hyperthermia [18]. However the expectation of late effects of higher cumulative doses limits the second treatment. Therefore, this analysis concerning re-irradiation to a median total dose of 60 Gy could facilitate a balanced treatment decision.

This series clearly demonstrates that the best long-term local control was achieved in patients treated with a combined schedule i.e. surgery and re-irradiation predominately performed with hyperthermia and combined with anti-hormonal treatment. Reducing the risk of local relapse using endocrine therapy is in line with findings concerning primary treatment of breast cancer [19,20]. Eighty percent of secondary recurrences after re-treatment occur within two years [21]. Therefore it is assumed that another recurrence is unlikely, and this assumption is supported by a median follow-up time of 48 months for survivors. Regarding the estimated five year overall survival rate of 75% in the combined treatment group, it is concluded that durable local control results in improved prognosis.

In 1999, the working group reported a three year local control rate of 80% for complete resections of breast cancer recurrences (most without pre-radiation) after a radiation schedule of 4 × 2.5 Gy/week to a total dose of 50 Gy. This resulted in local control of only 37% for R1/2-resections despite an additional boost to a total radiation dose of 60 Gy [22]. To intensify radiotherapy, concurrent hyperthermia was introduced leading to 81% local control after three years for marginally excised patients [23]. This series focused on a very unfavourable and rare subset of patients defined by breast cancer recurrence plus previous irradiation. Therefore, this analysis concerns a small number of patients with updated follow-up of the last mentioned investigation. However, even in cases of re-irradiation and dose compromise, for exceptional patients (R0-resection of recurrence plus pre-radiation exposure of 60 Gy) the referred local control rate could be replicated.

Probably as a result of manifold and overlapping parameters and small sample size of subgroups, local control and survival parameters were improved, but not significantly, by concurrent hyperthermia in this series. The role of hyperthermia after R0-resection remains unclear in this retrospective series. However, it can be assumed that additional hyperthermia compensated for positive margin (R1).

In the unfavourable situation of no surgical options, patients have the greatest incremental gain in complete response by additional hyperthermia according to a recently published randomized trial (23.5% vs. 68.2%) [24]. A pooled analysis of five poorly recruited randomized trials addressed this issue and estimated a 59% CR-rate for thermoradiotherapy compared with 41% for irradiation alone. Similarly, the greatest benefit was observed in patients with recurrent lesions in previously irradiated areas [8]. The data are comparable with the reported local control rates of other re-irradiation series+/-hyperthermia with 24-75% [5,7-9,25-28]. Furthermore, hyperthermia doubled overall survival (n.s.) in patients with residual disease.

A recently published multi-institutional review of re-irradiation achieved a 53% 1-year-disease-free survival for unresected patients. Hyperthermia and chemotherapy were applied simultaneously to more than half the women with a median total dose reaching 48 Gy [5]. Patients with gross residual disease did not receive additional chemotherapy in this study but did receive a higher median radiation dose. The calculated 1-year and estimated 5-year-disease-free survival of patients with gross residual disease were in the same range, 46% and 28%, respectively.

The only significant tumour-related parameter for overall survival was time below two years to first recurrence. This finding has been substantiated by others [17,29]. A trend to better survival for initially node-negative patients demonstrates the lower aggressiveness of the disease [30]. Favourable results could arise from the young median age of the patient population. Younger patients are more frequently affected by isolated recurrences [31]. This fact explains why re-surgery was possible and why the distant failure rate was relatively low compared to other studies [15].

In this series, one of the highest reported median re-irradiation- and cumulative radiation dose was administered. Owing to a conventionally fractionated regimen acute and early late toxicity was moderate as indicated by late effects not exceeding grade 3. This observation is consistent with other retrospective series that reach lower cumulative doses [27,32]. However, pronounced late toxicity was observed with a hypofractionated protocol for excised patients consisting of 2 × 4 Gy/week to a total dose of 32 Gy plus weekly hyperthermia. Fourty percent experienced late toxicity ≥grade 3 after three years [33]. Originally, this protocol was developed for palliation [34,35]. Nineteen percent (n = 8) grade 3 fibrosis and one case of rib fracture were detected after a median follow-up of 41 months. To evaluate late effects such as brachial plexopathy or cardiac failure further observation is required.

## Conclusions

Standard treatment for breast cancer recurrences is surgical resection, if possible. In general, owing to the detrimental effect of recurrences, early re-irradiation is recommended at first recurrence and a fortiori in case of recurrence within two years, as both factors were associated with dramatically reduced local control and overall survival.

Repeat radiotherapy for breast cancer recurrence, with total radiation doses of 60 Gy and the addition of hyperthermia in the majority of patients is feasible. This treatment has acceptable late morbidity and results in improved prognosis particularly in patients who have undergone previous resection of the recurrence.

## Competing interests

The authors declare that they have no competing interests.

## Authors' contributions

ACM: Substantial contributions to acquisition, analysis and interpretation of data, drafting the article and final approval. FE: Substantial contributions to interpretation of data, drafting and revising the article and final approval. VH: Substantial contributions to design, revising the article critically and final approval. MB: Substantial contributions to conception, revising the article critically and final approval. SB: Substantial contributions to interpretation, revising the article critically and final approval. TH: Substantial contributions to conception, analysis and interpretation of data, drafting the article and final approval.

## Pre-publication history

The pre-publication history for this paper can be accessed here:

http://www.biomedcentral.com/1471-2407/11/197/prepub
